# Point-of-care Lung Ultrasound Is Useful to Evaluate Emergency Department Patients for COVID-19

**DOI:** 10.5811/westjem.2020.8.49205

**Published:** 2020-09-28

**Authors:** Paul Walsh, Andrea Hankins, Heejung Bang

**Affiliations:** * Sutter Medical Center Sacramento, Department of Emergency Medicine, Sacramento, California; † Sutter Institute for Medical Research, Sacramento, California; ‡ University of California Davis, Department of Public Health Sciences, Davis, California

## Abstract

**Introduction:**

Coronavirus disease 2019 (COVID-19) can be a life-threatening lung disease or a trivial upper respiratory infection depending on whether the alveoli are involved. Emergency department (ED) evaluation of symptomatic patients with normal vital signs is frequently limited to chest auscultation and oro-nasopharyngeal swabs. We tested the null hypothesis that patients being screened for COVID-19 in the ED with normal vital signs and without hypoxia would have a point-of-care lung ultrasound (LUS) consistent with COVID-19 less than 2% of the time.

**Methods:**

We performed a retrospective, structured, blinded ultrasound review and chart review in patients 14 years or older with symptoms prompting ED evaluation for COVID-19. We excluded those with known congestive heart failure or other chronic lung conditions likely to cause excessive B-lines on LUS. We used a two-sided exact hypothesis test for binomial random variables. We measured LUS diagnostic performance using computed tomography as the gold standard.

**Results:**

We reviewed 77 charts; 49 met inclusion criteria. Vital signs were normal in 30/49 patients; 10 (33%) of these patients had LUS consistent with viral pneumonitis. We rejected the null hypothesis (p-value <0.001). The treating physicians’ interpretations of their own point-of-care LUS had a sensitivity of 100% (95% confidence interval (CI), 74%, 100%), specificity 88% (95% CI, 47%, 100%), likelihood ratio (LR) positive of 5.8 (95% CI, 1.3, 25), and LR negative of 0.05 (95% CI, 0.03, 0.71) when compared to CT findings.

**Conclusion:**

LUS had a meaningful detection rate for pneumonitis in symptomatic ED patients with normal vital signs who were being evaluated for COVID-19. We recommend at least LUS be used in addition to polymerase chain reaction testing when evaluating symptomatic ED patients for COVID-19.

## INTRODUCTION

Severe acute respiratory syndrome coronavirus 2 (SARS-CoV-2) causes a variety of respiratory symptoms ranging from pharyngitis or rhinitis, through bronchitis to multifocal peripheral pneumonitis extending to the alveoli.^1^–^3^ Two clinically important characteristics of SARS-CoV-2 infection are that auscultatory findings may be subtle or normal even in the presence of advanced lower airway disease, and chest radiographs (CXR) are inadequate for diagnosis.^4^ In common with other coronaviruses and influenza, SARS-CoV-2 is likely spread by both the droplet and airborne routes.^5^–^7^ When aerosolized, the resulting respirable particles less than 10 microns (μ) in aerodynamic diameter contain viable virus and can reach adult alveoli directly.^8^ Smaller aerosols (5μ) reach the alveoli without also being deposited in the bronchi.^8^ This can lead to a clinical picture where a patient has serious lower respiratory tract infection with little or no concomitant upper respiratory tract infection.^6^ Consequently, respiratory tract coronavirus disease 2019 (COVID-19) must be thought of as two separate entities. The first is upper airway disease, which generally poses little risk to the individual patient but places those around them at risk of infection. The second is lower airway disease where the patient is potentially at grave risk but who may shed little or no virus for much of his or her illness. These entities may coexist, but because transmission can occur by either the droplet or airborne routes, they may not. Nasopharyngeal swabs, even if correctly collected, can therefore fail to detect SARS-CoV-2 and provide false reassurance despite ongoing alveolar destruction.

Testing for SARS-CoV-2, therefore, frequently but not always includes both viral swabs from the oro-nasopharynx and imaging of the lower respiratory tract. This has included CXR, computed tomography (CT) imaging, and sometimes point-of-care lung ultrasound (LUS). Chest CT in the presence of lower respiratory tract involvement has a characteristic appearance and has been shown to be useful for diagnosing patients with COVID-19 pneumonia, including in the presence of negative nucleic acid testing. Some experienced centers even advocate CT imaging as a primary testing modality. However, CT imaging is slow, exposes the patient to ionizing radiation, and exposes additional staff to SARS-CoV-2.^4^,^9^

Point-of-care LUS can detect SARS-CoV-2-induced lung disease, is readily available in most emergency departments (ED), does not expose the patient to ionizing radiation, and does not require the staff, expertise, and time necessary for traditional CT imaging.^10^ Nonetheless, point-of-care LUS does add to the duration of patient evaluation, increases the treating physicians’ exposure to SARS-CoV-2, and decreases the number of patients seen hourly by that physician. This raises the question as to whether lung imaging could be deferred if the patient being evaluated for SARS-CoV-2 has normal vital signs. Conversely, if the presence of normal vital signs does not preclude ultrasound evidence of lung disease then some current practices of swab-only testing must be considered inadequate. Patents with lung involvement have been shown to be at risk for subsequent, sometimes rapid, deterioration.^9^ Patients are often not aware of this deterioration and attendant hypoxia. Consequently, such patients require at least home pulse oximetry.

Our null hypothesis was that among symptomatic patients being screened for COVID-19 in the emergency department (ED) that the LUS would be consistent with COVID-19 less than 2% of the time if vital signs were normal. We also measured the diagnostic performance of LUS compared with CXR and CT chest. For comparative purposes we also measured the diagnostic performance of CXR and crackles or rales on auscultation with CT chest.

## METHODS

### Ethical approval

The institutional review and privacy boards for Sutter Health approved this study and granted a waiver of informed consent (approval number 1597263).

**Population Health Research Capsule**

What do we already know about this issue?

*Auscultation and chest radiograph mostly fail to detect lung involvement in coronavirus disease 2019 (COVID-19)*.

What was the research question?

Do normal vital signs mean lung imaging is unnecessary when evaluating patients for COVID-19 in the ED?

What was the major finding of the study?

*In symptomatic patients with normal vital signs 33% had lung ultrasound (LUS) evidence of alveolar involvement*.

How does this improve population health?

*Point-of-care LUS can aid in risk stratifying symptomatic ED patients in whom COVID-19 is suspected*.

### Study Design

This was a cross-sectional study with structured chart and ultrasound imaging review.

### Subjects

Subjects were a consecutive sample of patients, 14 years of age and older, who received LUS and were evaluated for COVID-19 in an adult ED and a pediatric ED between March 4, 2020–May 19, 2020. We identified subjects from the imaging archive of the ED ultrasound machine. Patients had LUS performed if the treating physician was facile in point-of care LUS, presumably believed that lung imaging should form part of the COVID-19 evaluation, and did not send the patient for immediate CT of the chest.

### Ultrasound Imaging Protocol

The physicians performing the LUS typically imaged the posterior acoustic windows by running the ultrasound probe down the patient’s back midway between the scapula and vertebral column. Axillary and anterior windows were typically interrogated with single views of each. Physicians sometimes chose to not interrogate all possible windows if they had already reached their diagnosis on the windows already imaged. Images were captured with a Zonare Z One ULTRA portable ultrasound machine (Zonare Medical Systems, Mountain View, CA). The probes available for use were linear 10-5 megahertz (MHz), linear 4-1 MHz, and curvilinear 9-3 MHz. For our primary analysis we used the interpretation of the LUS as documented in the chart.

We also performed a second interpretation of the stored ultrasound images blinded to any clinical information and the original bedside interpretation. For this interpretation of the ultrasound images we considered the following findings to be consistent with viral pneumonitis: more than three simultaneous long coalescent B-lines per intercostal space occurring in more than one intercostal space; moth-eaten or irregular pleura in two or more interspaces or in one interspace with adjacent pleura showing excessive short B lines (comet tails). We considered A-lines, isolated short B-lines (comet tails) without adjacent moth-eaten pleura, and Z-lines (defined here as horizontal reverberation lines at a higher frequency than A lines) to be normal. Focal consolidations or effusions were taken as evidence against viral pneumonitis.

### Inclusion Criteria

We included subjects if they met the following criteria: they were 14 years of age or older; they had had ultrasound images archived with adequate identifiers; and they were being evaluated for SARS-CoV-2 infection causing a COVID-19 illness.

### Exclusion Criteria

Patients were excluded for a prior medical history of congestive heart failure, based on chart review, or other chronic lung disease likely to affect LUS interpretation (ie, disease likely to cause B lines or pleural thickening) and if the point-of-care LUS was performed for a reason other than evaluating for COVID-19. We did not exclude patients with a history of asthma or chronic obstructive pulmonary disease. Patients were also excluded if we could not pair the written record of their ED visit with the ultrasound images. This happened when ultrasound images were saved without identifiers.

### Study Definitions

We defined “symptomatic” as the documentation of any of the following in the electronic health record (EHR): cough; subjective fever; fatigue; weakness; sore throat or shortness of breath; nausea or vomiting; diarrhea; sore throat; fatigue; or headache. We defined “abnormal” vital signs as pulse or respiratory rate at or above the 98th percentile for age for children.^11^ For adults, tachycardia was defined as pulse at or above 100 beats per minute, tachypnea as respiratory rate above 22 breaths per minute, fever as temperature as ≥38° Celsius, and hypotension as systolic blood pressure at or below 80 millimeters of mercury (mm Hg).^12^ We did not have an upper limit for blood pressure. We included oxygen saturation measured by pulse oximetry as a vital sign and defined hypoxia as oxygen saturation of less than 92%.

We accepted the interpretation of the ultrasound by the performing physician as consistent with COVID-19 or viral pneumonitis for our primary analysis. On three occasions when the performing physician did not document an interpretation we substituted the blinded reading.

### Data Abstraction

One investigator (PW) performed a blinded reading of all LUS images using a structured template prior to performing chart review. Another (AH) extracted data from the EPIC/Clarity EHR (Verona, WI) using SQL Server Management Studio (Microsoft Corporation, Redmond, WA). Vital signs for each visit and polymerase chain reaction (PCR) results of swabs were extracted from their respective fields in the EHR. Only the first set of vital signs was retained. Vital signs and lab results were directly extracted from the EHR. The full text of the ED visit was downloaded into a text file. EPIC EHR periodically automatically saves even incomplete notes as they are entered. The time of each (even incomplete) note is recorded. This allowed us to ensure the ultrasound note was entered before the CT resulted.

The ultrasound note was typically entered either in free form or using personalized, physician-created templates. These were in various locations in the chart. Some were typed into distinct, stand-alone progress notes and others were included in the main chart, while still others were included in progress notes that included another patient’s information. We used a simplified sentiment analysis (sentimentr) in R (R Foundation for Statistical Computing, Vienna, Austria) to locate the bedside ultrasound report in the chart.^13^ This created an HTML page highlighting text that sentiment analysis considered to be an ultrasound report. In three cases a bedside ultrasound report could not be found using either this semi-automated technique or a manual chart review, and we substituted the blinded interpretation.

CT and CXR results have standardized headers and were located using regexm functions in Stata (StataCorp, College Station, TX) and then manually reviewed and data abstracted using a standardized template by an author (PW). Because there was only one chart reviewer, inter-rater reliability was not a concern. We did not attempt intrarater reliability measurement of the chart abstraction process.

### Data and Statistical Analysis

We tested the null hypothesis using the bitest command in Stata. This performs exact hypothesis tests for binomial random variables. The null hypothesis was that the probability of a positive ultrasound was 2%. Our sample size calculations are shown in Appendix 1. We compared inter-rater reliability between the treating physician and reader relying on only the archived images using Gwet’s agreement coefficient (AC1). The validity of Gwet’s AC1 does not depend upon the hypothesis of independence between raters and it does not result in unexpectedly low values (as seen in Cohen’s κ) when agreement is expected to be high.^14^,^15^ We have previously shown how Cohen’s κ can be misleading in pediatric emergency medicine research and why alternatives such as Gwet’s AC1 should often be used instead. 16 We used kappaetc in Stata to calculate Gwet’s AC1.^17^ We measured diagnostic performance of the point-of-care LUS using board-certified radiologists’ interpretations of the CT chest as the gold standard using the diagt command in Stata.^18^ Data and statistical analysis was performed using Stata 16.1 and R.

## RESULTS

We identified 77 point-of-care LUS with associated medical records of which 49 met our inclusion and exclusion criteria. All 77 scans were used to measure inter-rater reliability and diagnostic performance characteristics. All the point-of-care LUS were performed before the CTs. Figure shows patient flow through the study. The demographic characteristics of subjects are shown in Table 1.

The treating physician interpreted 18/49 (37%) point-of-care LUS as being consistent with COVID-19. Vital signs were normal in 30 patients, and 10 (33%) of these patients had LUS consistent with COVID-19. We therefore reject the null hypothesis that among symptomatic patients being screened for COVID-19 in the ED that the point-of-care LUS would be consistent with COVID-19 less than 2% of the time if vital signs were normal (p-value <0.001). We accept our alternative hypothesis that point-of-care LUS would be consistent with COVID-19 more than 2% of the time even if the vital signs were normal.

When compared with the subsequent CT, the treating physicians’ interpretation of their own point-of-care LUS had a sensitivity of 100% (95% confidence interval [CI], 74%–100%) and specificity of 88% (95% CI, 47%–100%). For the over-reading physician relying only on archived images the sensitivity and specificity were 92% (95% CI, 62%–100%) and 37% (95% CI, 25%,−50%), respectively. All but one of the CTs that were interpreted as positive reported multiple, ground-glass opacities. One CT report that did not explicitly report ground-glass opacities did report “bilateral interstitial changes” and an explicit radiology opinion that the CT lung appearance was consistent with COVID-19. The performance characteristics of point-of-care LUS using CT chest as the gold standard are detailed in Table 2.

Inter-rater agreement measured using Gwet’s AC1 between the bedside physician who performed the point-of-care LUS and the over-reading physician using only archives was 68%. Most characteristics showed acceptable inter-rater reliability between the bedside read and images that were over-read (Table 3). Excess short non-coalescent B-lines and pleural thickening showed poor agreement likely reflecting both the subjectivity of these items and the difference between reviewing saved and real-time images.

PCR testing for SARS-CoV-2 was not always available, but when it was a variety of tests performed at different sites were used. The results are shown in Table 4.

## DISCUSSION

LUS detected lesions consistent with alveolar involvement in 33% of symptomatic patients with normal vital signs who were being screened for COVID-19. A key underlying assumption of our work was that a negative nasopharyngeal swab does not exclude COVID-19. This assumption has been repeatedly shown to be valid with studies finding negative nasopharyngeal swabs but positive bronchoalveolar lavage for SARS-CoV-2, SARS-CoV-1, and Middle East respiratory syndrome.^19^–^21^

Our findings are consistent with published case series and social media reports of the utility of LUS in the diagnosis of COVID-19.^22^,^23^ The use of point-of-care LUS in COVID-19 evaluation has been spontaneous and sporadic practice typically occurring in emergency medicine and critical care. Some radiologists have also found LUS useful.^22^,^23^ Regardless of the specialty, point-of-care LUS practices in the detection of COVID-19 have necessarily evolved ahead of their published evidence base. The peer-reviewed literature is sparse. Previous literature has comprised case reports, and case series of 12 and 20 patients.^23^–^25^ Scanning techniques, and images of patients with proven COVID-19 have spread among clinicians on Twitter and blogs^26^,^27^ among others, and at least one COVID-19 ultrasound scoring system has been proposed.^28^

LUS has emerged as a clinical tool in human and veterinary medicine and in animal research with some advocates calling for it to replace the stethoscope.^29^–^32^ Others have shown ultrasound to complement rather than replace the physical exam and to correlate reasonably well with lung findings at necropsy. Ultrasound decreases CT utilization in inpatients with suspected COVID-19.^33^ Descriptive papers have found that ultrasound correlates well with CT and clinical characteristics in COVID-19 patients.^34^,^35^ Recommendations for training novices to identify COVID-19 have started to appear.^33^ Ultrasound cannot be expected to replace CT imaging; but the ease with which it can be performed serially, at the bedside, makes it a useful tool for detecting alveolar level disease in SARS-CoV-2 infection.

We believe that knowing whether a patient has alveolar involvement with COVID-19 is clinically important. Patients’ initially mild lung disease has been shown to progress, sometimes rapidly, on serial CTs as the disease progresses.^36^ LUS does give a semi-quantitative estimate of how extensive the lung involvement is. When the lung is not involved discharge is likely safe. When there is only mild lung disease and vital signs are normal our practice is to discharge these patients with a home pulse oximeter. But if ultrasound shows that the patient has widespread pneumonitis then he or she should be investigated further. Patients frequently are unaware of their own deterioration and may present, or fail to re-present with critically low oxygen saturation without overt symptoms. These patients frequently have negative PCR tests unless bronchoalveolar lavage is performed. Such patients risk being falsely reassured about their own impending fate, and continue to infect others when, inevitably, they cough.

## LIMITATIONS

This was a single-center study and was not a random sample. Whether a patient was seen by a physician who both believed that the COVID-19 evaluation should include lung imaging and was facile with ultrasound was a matter of luck rather than randomization. This adds uncertainty to estimates of the prevalence of pneumonitis that point-of-care LUS can detect among patients being screened for COVID-19. Other limitations of our work include its small sample size, and a single chart reviewer. Patients with mild disease, and especially those with normal vital signs, did not always have CT imaging performed. PCR testing for SARS-CoV-2 was not always available; and even when PCR testing was available, the gold standard of bronchoalveolar lavage to obtain a specimen was not performed.

Our use of CT as a gold standard is imperfect as CT diagnosis of COVID-19 has its own limitations.^37^ It is difficult to conceive of an alternative gold standard that does not fall afoul of circular reasoning (by, for example, using “two out of three” imaging methods positive as the gold standard). Another limitation is that CT was likely reserved for patients perceived as being sicker or having more extensive lung disease on ultrasound. This could have created a spectrum bias that would have increased the apparent accuracy of LUS. However, CT cannot be justified on patients simply to better determine the test characteristics of LUS. Finally, because of the false negative rates of PCR testing, CT rather than PCR testing has been recommended as the primary diagnostic modality in high prevalence settings.^38^

Our assessment of the performance characteristics of ultrasound is limited by our sample size. The relative subjectivity of LUS is also a limitation. We observed much less agreement between the blinded reviewer looking only at ultrasound images and the treating physician performing the LUS. We speculate that pleural findings were more subjective and the decision that pleural findings were abnormal might have been influenced by the clinical picture. However, describing the performance characteristics of LUS was not the primary aim of this study. Although falling out of favor, null hypothesis testing is well suited to answering our primary question when the sample size is small – after all, a single “red” (brown) Holstein cow demolishes the hypothesis that all cows are black and white, and careful planning minimizes the number of cows that need to be seen.

Despite these limitations, we can be assured that the prevalence of pneumonitis in these patients was more than the 2% “acceptable miss rate” for high morbidity conditions, and this may be sufficient to adjust practice accordingly.^39^ Other limitations include the use of abbreviated LUS imaging protocols and the variability in image-saving practices with some doctors saving many cine-clips, while others saved only one or two still images. These differences in practice style could decrease inter-rater agreement between the blinded and bedside readings. Much more detailed and formalized LUS protocols and ultrasound scoring systems specifically for use in SARS-CoV-2 patients have been described.^28^,^35^ Abbreviated protocols are inevitable in community practice and could lead to missed diagnoses. This would have biased our study in the opposite direction of our actual findings.

## CONCLUSION

In this small, single-center study, point-of-care lung ultrasound had a meaningful detection rate for pneumonitis in symptomatic ED patients with normal vital signs who were being evaluated for COVID-19. Test characteristics were as follows: sensitivity 100%; specificity 88%; PPV 92%; NPV 100%; LR+ 5.8; and LR− 0.1 with broad confidence intervals when compared to CT. We recommend at least point-of- care lung ultrasound be used in addition to PCR testing to identify lower airway disease when evaluating symptomatic patients in whom SARS-CoV-2 infection is suspected.

## Supplementary Information







## Figures and Tables

**Figure:**
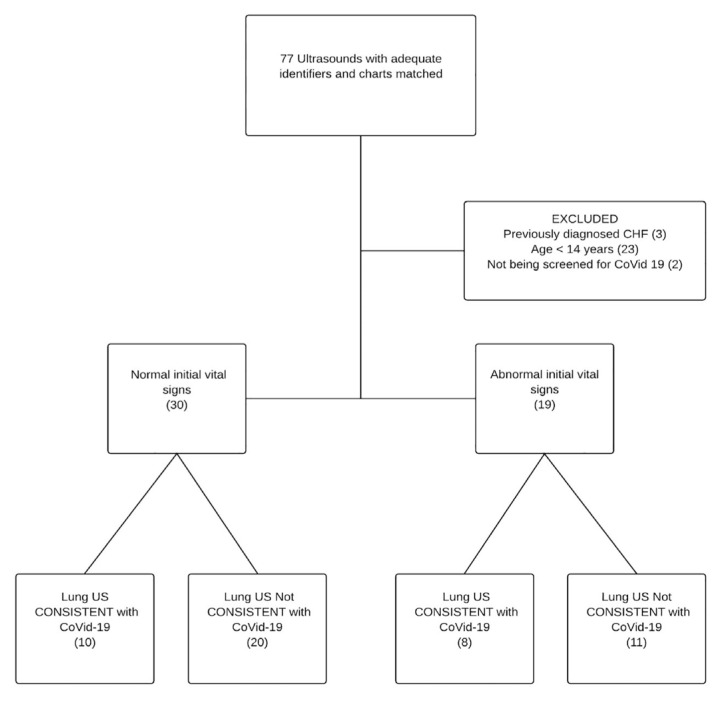
This figure shows patient flow through the study. Given the clinical context of evaluating suspected COVID-19 the presence or absence of lung ultrasound findings consistent with viral pneumonitis was interpreted as consistent with COVID-19. *COVID-19*, coronavirus disease 2019; *US*, ultrasound; *CHF*, congestive heart failure.

**Table 1:** Clinical characteristics of study patients overall, and the presence or absence of lung ultrasound findings consistent with viral pneumonitis.

		Total (N=49)	LUS not suggestive of viral pneumonitis (N=31)	LUS suggestive viral pneumonitis (N=18)
Gender	Male	25(51%)	13(42%)	12 (67%)
Age (years)	Median (IQR)	25 (15–46)	22 (14–52)	31 (16–46)
Duration (days)	Median (IQR)	4 (2–7)	3 (2–7)	5(3–8)
Subjective fever at home	Present	16 (33%)	9 (29%)	7 (39%)
Cough	Present	26 (53%)	15 (48%)	11 (61%)
Dyspnea	Present	29 (59%)	18 (58%)	11 (61%)
Sore throat	Present	9 (18%)	7 (23%)	2 (11%)
Fatigue	Present	9 (18%)	6 (19%)	3 (17%)
Headache	Present	14 (29%)	7 (23%)	7 (39%)
Myalgias	Present	5 (10%)	4 (13%)	1 (6%)
Diarrhea	Present	6 (12%)	2 (6%)	4 (22%)
Nausea/vomiting	Present	8 (16%)	5 (16%)	3 (17%)
Vital signs	Abnormal	30 (61%)	20 (65%)	10 (56%)
Tachycardia	Tachycardia	14 (29%)	10 (32%)	4 (22%)
Tachypneic	Tachypneic	4 (8%)	2 (6%)	2 (11%)
Hypotension	Normotensive	49 (100%)	31 (100%)	18 (100%)
Hypoxic	Hypoxia	5 (10%)	2 (6%)	3 (17%)
Lungs clear on auscultation	Present	35 (71%)	23 (74%)	12 (67%)
Crackles/rales on auscultation	Present	4 (8%)	3 (10%)	1 (6%)
Wheezing/ronchi on auscultation	Present	6 (12%)	3 (10%)	3 (17%)

*LUS*, lung ultrasound; *IQR*, interquartile range.

**Table 2:** Comparison of diagnostic performance of bedside point-of-care lung ultrasound, chest radiograph, and crackles on auscultation for diagnosis of lung involvement of SARS-CoV-2 using CT chest as the gold standard. These diagnostic performance characteristics are applicable only in the context of a patient who is symptomatic and was being specifically evaluated for COVID-19. Patients with known chronic heart failure and chronic lung disease, apart from asthma, have been excluded.

	Sens %	95% CI	Spec %	95% CI	PPV %	95% CI	NPV %	95% CI	LR+	95% CI	LR−	95% CI	AUC	95% CI
Modality														
Ultrasound	100	74–100	88	47–100	92	64–100	100	93–100	5.8	1.3–25	0.1	0.0–0.7	0.94	0.82–0.99
Chest radiograph	25	5–57	88	47–100	75	19–99	44	20–70	2.0	0.3–16	0.9	0.6–1.3	0.56	0.39–0.74
Crackles/rales	8	0–38	71	29–96	33	1–91	31	11–59	0.3	0.0–3	1.3	0.8–2.1	0.40	0.20–0.60

*Sens*, sensitivity; *CI*, confidence interval; *Spec*, specificity; *PPV*, positive predictive value; *NPV*, negative predictive value; *LR+*, likelihood ratio positive; *LR−*, likelihood ratio negative; *AUC*, area under the receiver-operating characteristic curve.

**Table 3:** Inter-rater agreement between a blinded over-read relying only on saved images and the bedside interpretation of the treating physician. Where the readings differed, the interpretation of the bedside physician ultrasonographer was used.

Ultrasound finding	% Agreement	95% CI	Gwet AC_1_	95% CI
Normal study	71	59–82	0.44	0.22–0.66
Excess coalescent (long) B lines	75	65–85	0.51	0.31–0.71
Excess short B lines (comet tail)	55	43–66	0.15	−0.10–0.39
Effusion	91	84–97	0.90	0.81–0.98
Air bronchograms	69	58–79	0.51	0.31–0.72
Thickened/moth-eaten pleura	53	42–65	0.11	−0.13–0.35
Atelectasis	69	58–79	0.51	0.31–0.71
Consolidation	80	71–90	0.74	0.60–0.88

*CI*, confidence interval; *AC_1_*, agreement coefficient.

**Table 4:** PCR results from nasal, nasopharyngeal, and oropharyngeal swabs, and lung ultrasound results. Although the overall number of polymerase chain reaction tests was the same, some patients received SARS-CoV-2 testing alone, while others had a panel of respiratory pathogens ordered without SARS-CoV-2 due to lack of test availability at the time. The panel of respiratory pathogens tested included adenovirus, parainfluenza viruses 1–4, *Mycoplasma pneumoniae*, *Bordetella pertussis*, coronaviruses 229E, HKU1, N163 and OC43; respiratory syncytial virus; human metapneumovirus; *Chlamydophila*; and *Chlamydophila pneumoniae*.

	PCR testing positive (%)	PCR testing negative (%)	US consistent with viral pneumonitis (%)	US not consistent with viral pneumonitis (%)
N = 49			18/49 (37)	31/49 (63)
Testing performed (N =42)			17/18 (94)	25/31 (81)
SARS CoV-2	5(12)	37 (88)	4 (24)	1 (4)
Influenza A	1 (2)	41 (98)	0 (0)	1 (4)
*Chlamydophila*	1 (2)	41 (98)	0 (0)	1 (4)

*PCR*, polymerase chain reaction; *US*, ultrasound; *SARS CoV-2*, severe acute respiratory syndrome coronavirus 2.
