# Supplementary figures and images for: Point-of-care Lung Ultrasound Is Useful to Evaluate Emergency Department Patients for COVID-19
Source: West J Emerg Med. 2020 Sep 28;21(6):24–31. doi: 10.5811/westjem.2020.8.49205 (PMC7673866; doi:10.5811/westjem.2020.8.49205)

## Simulated -bitest- p-values for various possible outcomes

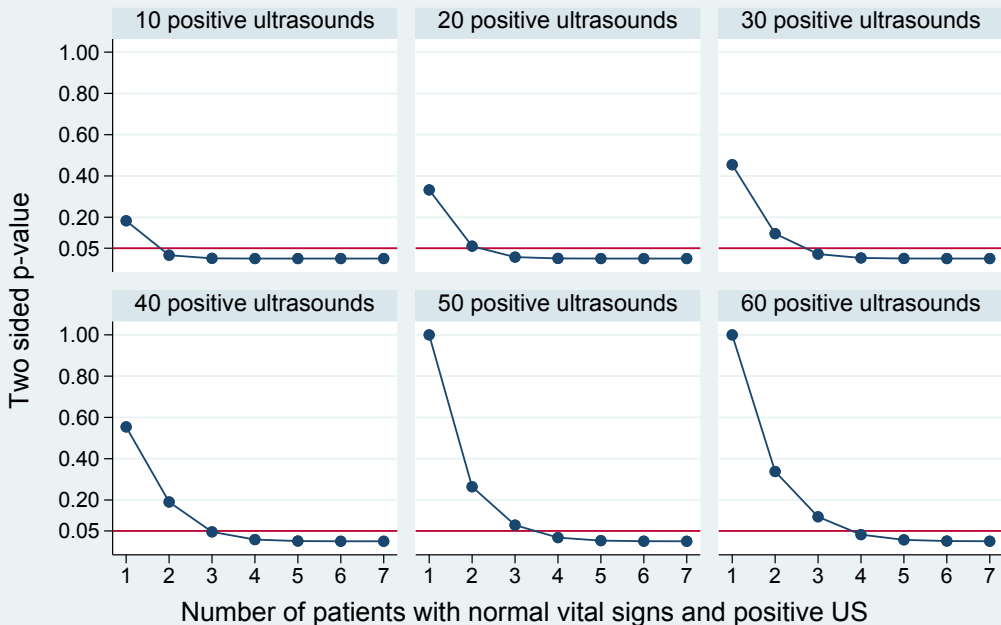

Supplement: Supplementary file 1 [file wjem-21-24-s001.pdf]
