# Supplementary material for: Point-of-care Lung Ultrasound Is Useful to Evaluate Emergency Department Patients for COVID-19
Source: West J Emerg Med. 2020 Sep 28;21(6):24–31. doi: 10.5811/westjem.2020.8.49205 (PMC7673866; doi:10.5811/westjem.2020.8.49205)
Supplement: Supplementary file 3 [file wjem-21-24-s003.pdf]

```
///
// Replicates whole paper  H: drive is a virtual drive on my sutter machine
//  put in your own drive here or it won't work

use "H:\Covid\PUD_COVID.dta"  ,clear


lab var age "Age in years"


// Sort out gender


//Define as symptomatic

cap drop symptomatic
cap label drop symptom
/*

destring temp, replace
destring pulse, replace
destring rr, replace
destring sbp ,replace
destring spo2, replace
*/

//cap drop age_check
//ge age_check =(dos-dob)/365
// Exclusion criteria////
cap drop exclude
cap lab drop exclude

gen exclude =0

    replace exclude =3 if age_check <14 // **Under 2s Dropped already exceluded by data steward**
    replace exclude =1 if hx_chf =="Yes"
    replace exclude =2 if hx_other_lung=="Yes"
    replace exclude =4 if temp >38.0 & antipyr_home =="Yes" & antipyr_hours <4
    replace exclude =5 if not_being_screened_for_covid =="Yes"

lab def exclude 0 "Included" 1"Prior CHF" 2 "Prior Lung disease" 3 "Age < 14 years" 4 "Confounding use of antypyretic" 5 "Not being screened for CoVid 19"

lab val exclude exclude


// Define abnormal for each then any
// define hypoxia
cap drop hypoxia
cap lab drop hypoxia
destring sa02, replace
gen hypoxia =1 if sa02 <=92
 replace hypoxia = 0 if sa02>92  & sa02 <.

// Define tachycardia
```

```
cap drop tachycardia
cap lab drop tachycardia
lab def tachycardia 1 "Tachycardia" 0 "Normal HR"

gen tachycardia =1 if hr >100 & age_check >=18 & hr !=.
 replace tachycardia =1 if hr > 105 & age_check >=14 & age_check <18 & hr !=.
 replace tachycardia =1 if hr > 120 & age_check >=5 & age_check <14 & hr !=.
 replace tachycardia = 0 if tachycardia==.

lab val tachycardia tachycardia

//Define hypotension
cap drop hypotensive
cap lab drop hypotensive
lab def hypotensive 1 "Hypotensive" 0 "Normotensive"

gen hypotensive =1 if sbp <=80 & age_check >18
 replace hypotensive =1 if sbp <80 & age_check >=14 & age_check<18
 replace hypotensive = 0 if hypotensive ==.
lab val hypotensive hypotensive

//Define febrile
cap drop febrile
cap lab drop febrile
lab def febrile 0 "Afebrile"  1 "Febrile"
 gen febrile =1 if temp >=100.4 & temp <.
 replace febrile =0 if temp <100.4
lab val febrile febrile

//define tachypnea

cap drop tachypnea
cap lab drop tachypnea
lab def tachypnea 0 "Normal RR " 1 "Tachypneic"

gen tachypnea =1 if rr >22 & age_check >=18
    replace tachypnea =1 if rr > 20 & age_check >=14 & age_check <18
    replace tachypnea =1 if rr > 30 & age_check>=5 & age_check<14
    replace tachypnea= 0 if tachypnea==. & rr <.
lab val tachypnea tachypnea

//
 cap drop vs_norm
 cap lab drop vitals
 lab def vitals  1 "Normal VS" 0 "Abnormal VS"
 gen vs_norm =1 if febrile+tachycardia +tachypnea +hypotensive + hypoxia ==0
 replace vs_norm =0 if  vs_norm ==. & ((febrile+tachycardia +tachypnea + hypotensive + hypoxia)  >0) & ((febrile+tachycardia +tachypnea +hypotensive + hypoxia ) <.)
 lab val vs_norm vitals

// Ho  That (among symptomatic patients being screened for Covid) Lung POCUS is never (<2%) normal.

// Ha That (among symptomatic patients being screened for Covid) Lung POCUS maybe abnormal (= Cw_covid)
```

```
//Actually testing the Null: Among patients with a Lung POCUS c/w Covid vital signs are never (<2% of cases normal)
// Actual Ha Among patients with a Lung POCUS c/w Covid   vital signs may be  abnormal >2% of the time.

// Variables needed for priamry outcome 1. norm vitals 2. US C/W covid  3. Total N of abnormal US These variables are
// vs_norm , norm_bin  , cw_covid

// Vitals_normal

 // US not enterd i dbase - now in dbase but don't want ot start over
 //replace normal = "No" if csn =="xx"// see original
// replace normal = "No" if csn =="xx"//see original



// Total number of US to include in N
cap drop us_pos_covid
gen us_pos_covid =.
replace us_pos_covid =1 if regexm(md_covid ,"Yes")
replace us_pos_covid =1 if regexm(md_covid ,"Yes")
replace us_pos_covid =0 if regexm(md_covid ,"No")


tab us_pos_covid  ,mis


lab var us_pos_covid "N (US c/w CoVid) for bitest"

// Alternatively for non-immediate form of test
bitest us_pos_covid == 0.02 if vs_norm==1&  exclude==0 ,detail


//
// Ct pos variable
cap drop pos_ct_covid_1
gen pos_ct_covid_1 =.
replace  pos_ct_covid_1  =0 if regexm(ct_covid , "No")
replace  pos_ct_covid_1  =1 if regexm(ct_covid , "Yes")
diagt pos_ct_covid_1 us_pos_covid  ,sf

//
//
cap drop over_read_us_pos
gen  over_read_us_pos  =1 if normal =="No" |regexm(normal, "No - Single image only")
replace over_read_us_pos =0 if over_read_us_pos ==.

//cap drop age_check
//format %td dob
//gen age_check  = (dos -dob)/365.25
cap drop age1

/* Figure 1  Initial N will be  _N
                Exclusions will be exclude 1-5

```

```
// Table 1
//Gender
// Age
// Day
*/
cap drop antipyretic_lt6
gen antipyretic_lt6=0  if antipyr_home =="Yes"
replace antipyretic_lt6 =1 if antipyr_hours <=6


//cough dyspnea subj_fever meas_fever duration antipyr_home antipyr_hours ausc_clear ausc_crackles aus_wheez_ronc hx_chf hx_other_lung cw_covid not_being_screened_for_covid sore_throat fatigue headache myalgias diarrhea vomiting exp_known_case
cap lab drop present_absent
lab def present_absent  0 "Absent" 1 "Present"

foreach var of varlist cough dyspnea subj_fever meas_fever  ausc_clear ausc_crackles aus_wheez_ronc hx_chf hx_other_lung  sore_throat fatigue headache myalgias diarrhea vomiting cxr_covid ct_covid{

    cap drop `var'_bin
    gen `var'_bin =1 if `var'=="Yes"
    replace `var'_bin = 0 if `var' =="No" | `var' ==""
    lab val `var'_bin present_absent
}


cap drop antipyr_home_bin
encode antipyr_home ,gen(antipyr_home_bin)

//label for table  cough_bin dyspnea_bin subj_fever_bin meas_fever_bin ausc_clear_bin ausc_crackles_bin aus_wheez_ronc_bin hx_chf_bin hx_other_lung_bin sore_throat_bin fatigue_bin headache_bin myalgias_bin diarrhea_bin vomiting_bin
cap lab drop hypoxia
lab def hypoxia  0 "Normoxia" 1 "Hypoxia"
lab val hypoxia hypoxia

lab var cough_bin "Cough"
lab var dyspnea_bin "Dyspnea"
lab var subj_fever_bin "Subjective fever at home"
lab var meas_fever_bin "Measured fever at home"
lab var ausc_clear_bin "Lungs clear on auscultation"
lab var ausc_crackles_bin "Crackles/Rales on auscultation"
lab var aus_wheez_ronc_bin "Wheezing or ronchi on auscultation"
lab var sore_throat_bin "Sore throat"
lab var fatigue_bin "Fatigue"
lab var headache_bin "Headache"
lab var myalgias_bin "Myalgias"
lab var diarrhea_bin "Diarrhea"
lab var vomiting_bin "Nausea/vomiting"
lab var hypoxia "Hypoxic"
lab var tachycardia "Tachycardic"
lab var tachypnea "Tachypneic"
lab var hypotensive "Hypotenison"
 table1_mc if exclude  ==0  ,vars(gender  cate\ age_check conts\ duration conts\  subj_fever_bin  cate\  cough_bin  cate\ dyspnea_bin cate\
```

```
subj_fever_bin cate\ sore_throat_bin cate\ fatigue_bin cate\  headache_bin
  cate\ myalgias_bin cate\ diarrhea_bin cate\ vomiting_bin cate\  vs_norm
cate\tachycardia cate\ tachypnea  cate\  hypotensive cate\ hypoxia cate \
ausc_clear_bin  cate\ausc_crackles_bin  cate\ aus_wheez_ronc_bin  cate ) by
(us_pos_covid)  total(before) saving(Table_1_covid_by_US.xlsx ,replace)




  table1_mc if exclude  ==0  ,vars(gender  cate\ age conts\ duration  conts
\  subj_fever_bin  cate\  cough_bin  cate\ dyspnea_bin cate\subj_fever_bin
cate\ sore_throat_bin cate\ fatigue_bin cate\  headache_bin  cate\
myalgias_bin cate\ diarrhea_bin cate\ vomiting_bin cate\ ausc_clear_bin
cate\ausc_crackles_bin  cate\ aus_wheez_ronc_bin  cate ) by(us_pos_covid)
total(before)

/* Next steps
    1. Compare performance of CXR and Lung POCUS versus CT with CT as
the   criterion refenrece(Gold standard)

    2. Compare inter rater reliability between live read and over-read
-blinded

    3. Compare inter rater reliabilty between two over readers

*/
 //  1. comparison of modalities

//// Diagt output

cap frame drop sens
frame create sens   modality sen  sen_lb sen_ub spec spc_lb spec_ub ppv
ppv_lb ppv_ub npv npv_lb npv_ub lrpos lrpos_lb lrpos_ub lrneg lrneg_lb
lrneg_ub  auc auc_lb auc_ub

foreach var of varlist us_pos_covid   cxr_covid_bin ausc_crackles_bin {

 diagt pos_ct_covid_1  `var'  ,sf0

frame post sens (`var') (r(sens)) (r(sens_lb)) (r(sens_ub))  (r(spec)) (r(
spec_lb)) (r(spec_ub)) ( r(ppv)) (r(ppv_lb)) (r(ppv_ub)) (r(npv)) (r(npv_lb
)) (r(npv_ub))  (r(lrp))  (r(lrp_lb)) (r(lrp_ub)) (r(lrn)) (r(lrn_lb)) (r(
lrn_ub))  (r(roc)) (r(roc_lb)) (r(roc_ub))

}

frame sens :save  table2_covid_pud.dta, replace


 //  Inter rater reliabilty

 //kappaetc pos_ct_covid_1 cxr_covid_bin

 //kappaetc pos_ct_covid_1 cw_covid

 //kappaetc ct_nil_acut_pulm mdUS_normalcopy_bin
```

```
 ///
foreach var of varlist excess_long_b excess_short_b thickened_pleura
effusion consolidation atelectasiss normal air_bronchograms
excess_long_bcopy excess_short_bcopy thickened_pleuracopy effusioncopy
consolidationcopy atelectasisscopy normalcopy air_bronchogramscopy {

    cap drop n_`var'
    gen n_`var' =1 if `var' =="Yes"
    replace n_`var' = 0 if `var' =="No"  |`var' ==""



}

foreach var of varlist excess_long_b excess_short_b thickened_pleura
effusion consolidation atelectasiss air_bronchograms  normal{

   di "__________________________________"
   di "__________________________________"

   di "`var'"


    kappaetc n_`var'  n_`var'copy

     di "`var'"

}



/* References

     bitest stata manual, Hoel  Mathematicla Statisitics 5Ed Wiley,

     table1   Phil Clayton, ANZDATA Registry, Australia, phil@anzdata.org.au


     diagt  Paul T Seed       (Paul.Seed@kcl.ac.uk)
        Maternal & Fetal Research Unit, GKT School of Medicine, KCL
        North Wing, St Thomas' Hospital, Lambeth Palace Road,
        London SE1 7EH

   kappaetc  Daniel Klein
    International Centre for Higher Education Research Kassel
    Kassel, Germany
    klein@incher.uni-kassel.de

*/
```
